# Rare Variant Association Testing by Adaptive Combination of *P*-values

**DOI:** 10.1371/journal.pone.0085728

**Published:** 2014-01-15

**Authors:** Wan-Yu Lin, Xiang-Yang Lou, Guimin Gao, Nianjun Liu

**Affiliations:** 1 Institute of Epidemiology and Preventive Medicine, College of Public Health, National Taiwan University, Taipei, Taiwan; 2 Department of Biostatistics, University of Alabama at Birmingham, Birmingham, Alabama, United States of America; 3 Department of Biostatistics, Virginia Commonwealth University, Richmond, Virginia, United States of America; University of North Carolina, United States of America

## Abstract

With the development of next-generation sequencing technology, there is a great demand for powerful statistical methods to detect rare variants (minor allele frequencies (MAFs)<1%) associated with diseases. Testing for each variant site individually is known to be underpowered, and therefore many methods have been proposed to test for the association of a group of variants with phenotypes, by pooling signals of the variants in a chromosomal region. However, this pooling strategy inevitably leads to the inclusion of a large proportion of neutral variants, which may compromise the power of association tests. To address this issue, we extend the 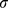 *-MidP* method (Cheung et al., 2012, Genet Epidemiol 36: 675–685) and propose an approach (named ‘adaptive combination of *P*-values for rare variant association testing’, abbreviated as ‘*ADA*’) that adaptively combines per-site *P*-values with the weights based on MAFs. Before combining *P*-values, we first imposed a truncation threshold upon the per-site *P*-values, to guard against the noise caused by the inclusion of neutral variants. This *ADA* method is shown to outperform popular burden tests and non-burden tests under many scenarios. *ADA* is recommended for next-generation sequencing data analysis where many neutral variants may be included in a functional region.

## Introduction

Next-generation sequencing acts as a new approach to explore the genetic basis of complex human diseases [1]. With this new technology, we are able to identify rare causal variants (minor allele frequency (MAF)<1%) that are not genotyped in genome-wide association studies (GWAS) but are actually responsible for part of the heritability of complex diseases. However, the power of an association test is largely compromised by the low frequencies of rare causal variants. To increase the power of an association test, many methods have been proposed to test for the collective effect of a group of variants in a chromosomal region [2]–[11]. These methods can be categorized as burden tests and non-burden tests.

Burden tests pool signals of multiple rare variants within a functional unit, such as a candidate gene, and then test for the association between the pooled signal (usually called “genetic score”) and the phenotype [2]–[5], [12]. In the Combined Multivariate and Collapsing (referred to as “*CMC*”) method, a subject's genetic score is defined as 1 if he/she has at least one rare variant in the gene and 0 otherwise [2]. The weighted-sum approach (referred to as “*WS*”) sums up the variant counts that are inversely weighted by the standard deviations of the variant frequencies [3]. Morris and Zeggini proposed to construct a genetic score by accumulating the variant counts in a functional unit (say, a gene or a pathway) [4], which was a variant of the *CMC* method. If only the counts of variants with frequencies smaller than 5% (or 1%) are aggregated as the genetic score, the test is referred to as “*T5*” (or “*T1*”). The threshold to discriminate rare variants from common variants is crucial, but the optimal threshold varies with the underlying genetic architecture and changes across studies [12]. The variable threshold (referred to as “*VT*”) approach was therefore proposed without a preset threshold. Instead, it searches for the optimal threshold that maximizes the difference between trait distributions for subjects with and without rare variants [5]. The above methods (including *CMC*, *T1*, *T5*, *WS*, and *VT*) are categorized as “burden tests”. These burden tests are more powerful when rare causal variants in a region have effects on the phenotype in the same direction, i.e., all are deleterious or all are protective [13].

On the other hand, non-burden tests, such as the so-called C-alpha test [9] or the sequence kernel association test (*SKAT*) [7] based on a kernel machine regression framework, are more robust to the inclusion of causal variants with disparate or even opposite effects on phenotype (we consider *SKAT* as a representative method of the non-burden tests, because it is a generalization of the C-alpha test). However, the non-burden tests such as *SKAT* can be less powerful than the burden tests if a large proportion of rare variants are associated with the phenotype in the same direction [13]. Because the underlying genetic function of a region is usually unknown, choosing an ideal statistical test (burden tests or *SKAT*) in advance is impossible. To develop a powerful test that is also robust to the directions of effects of rare variants, Lee et al. [8] have proposed an optimal test to combine *SKAT* [7] and the burden tests [2]–[5], [12]. This optimal test (referred to as “*SKAT-O*”) has been shown to outperform the burden tests and *SKAT* in a wide range of scenarios [8].

Both the burden tests and the non-burden tests suffer from power loss with the inclusion of neutral variants. A preferable method to analyze next-generation sequencing data should have the robustness to this type of noise. To this end, Cheung et al. [14] proposed a 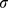 *-MidP* method that combines *P*-values of individual variants with the weighting scheme proposed by Madsen and Browning [3]. To guard against the noise caused by neutral variants, the 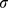 *-MidP* method excludes the variants with equal rare-variant counts in cases and in controls. Furthermore, 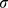 *-MidP* uses the Fisher's combination of *P*-values [15] on individual variants with the Madsen and Browning's [3] weighting scheme. This method has been shown to be more powerful than many existing methods [3]–[7], [9], [16], [17], when both deleterious and protective variants, or a large proportion of neutral variants, are present in a region [14].

Instead of testing for the association of a genetic score (some linear combination of variant counts) with the phenotype, 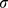 *-MidP*, inspired by the Fisher's combination of *P*-values, can take the significance of each variant site into account. To simplify, in the following small example we discuss the Fisher's combination method (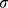 *-MidP* further uses the Madsen and Browning's [3] weighting scheme to facilitate the discovery of rare causal variants). Suppose there are *K* variants in a region of interest, the *P*-values of the *K* single-variant tests are combined with the Fisher's statistic: 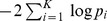 [15]. If there is a causal variant with a *P*-value of 0.05, it contributes 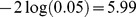 to the Fisher's statistic. However, the contribution to the Fisher's statistic will be only 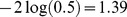 for a neutral variant with a *P*-value of 0.5. Because the *P*-values of causal variants are usually smaller than those of neutral variants, the contribution from causal variants to the Fisher's statistic is usually more prominent than that of neutral variants. Thus, different from testing the genetic score after summing variant counts (including causal variants and neutral variants), combining *P*-values after association testing can strengthen the association signal and guard against the noise caused by neutral variants.

To more effectively guard against the noise caused by neutral variants, variants with *P*-values larger than a threshold (they are more likely to be neutral) may be truncated (see [18] for the methodology and [19] for its application). However, the *P*-value truncation threshold of 0.05 (used in [19]) may be too stringent, because testing for each rare variant is usually underpowered [2], [20]–[22]. For rare variants detection, there is no general rule to choose a more “suitable” *P*-value truncation threshold. To address this issue, we here propose to determine the truncation threshold adaptively. Therefore, this method is termed *ADA* (full name: adaptive combination of *P*-values for rare variant association testing), which is inspired by the adaptive combination of *P*-values for pathway analysis in GWAS [23]. Instead of fixing a *P*-value truncation threshold, the proposed method allows multiple candidate truncation thresholds (say, 0.10, 0.11, 0.12, …, 0.20) and works out the optimal threshold for a given data set. The significance of our test is quantified with permutations. Comprehensive simulation studies indicate that the *ADA* method has a higher power than 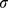 *-MidP* [14]. It also outperforms some popular approaches, including the burden tests such as *T1*, *T5*, *WS*, *VT* mentioned above, *SKAT* [7], and *SKAT-O* [8]. As an application, the data set from Dallas Heart Study [24], [25] is analyzed with the proposed method.

## Materials and Methods

Suppose there are *K* variants in a region of interest, and the *P*-values of testing for the associations of individual variants with the disease status are 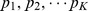, respectively. Without loss of generality, although we here focus on binary traits, the proposed method can be applied to continuous traits as well. In rare variants detection for binary traits, 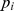
's are commonly obtained by the Fisher's exact test [14], [26]. Suppose we consider *J* candidate truncation thresholds on per-site *P*-values, 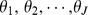. We term the sites with larger variant frequencies in cases than in controls “deleterious-inclined variant sites”. Among the *K* sites, the significance score of the deleterious-inclined variant sites is
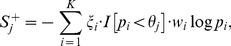
(1)where 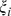 is an indicator variable coded as 1 if the *i*th site is deleterious-inclined and 0 otherwise, 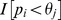 is an indicator variable coded as 1 if the *i*th site has a *P*-value smaller than 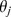 (the *j*th truncation threshold) and 0 otherwise, and 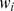 is a weight given to the *i*th site. Following Madsen and Browning [3], we specify 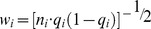, where 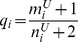 is the frequency for variant *i* in the unaffected individuals, 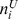 is the number of unaffected individuals genotyped for variant *i*, and 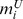 is the number of mutant alleles observed for variant *i* in the unaffected individuals [3]. We recommend using *J* = 11 candidate truncation thresholds, and we specify 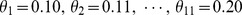 throughout this study (we will discuss the selection of candidate truncation thresholds in the Discussion section).

On the other hand, we term the sites with larger variant frequencies in controls than in cases “protective-inclined variant sites”. Among the *K* sites, the significance score of the protective-inclined variant sites is
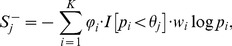
(2)where 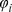 is an indicator variable coded as 1 if the *i*th site is protective-inclined and 0 otherwise. From Equations (1) and (2), we obtain the significance score accumulated by deleterious-inclined variants (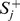) and that accumulated by protective-inclined variants (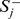), respectively. A test statistic regardless of the effect directions (deleterious or protective) is 
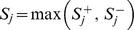
.

Because variant sites within a functional region are usually not independent, we need permutations to obtain the *P*-value of the observed statistic 
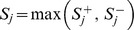
, for *j* = 1, …, *J*. For the *b*th permutation (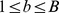), we randomly shuffle the case/control status and obtain 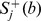 and 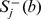 according to Equations (1) and (2). Then, we obtain the statistic 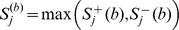, for *j* = 1, …, *J*.

**Figure 1:**
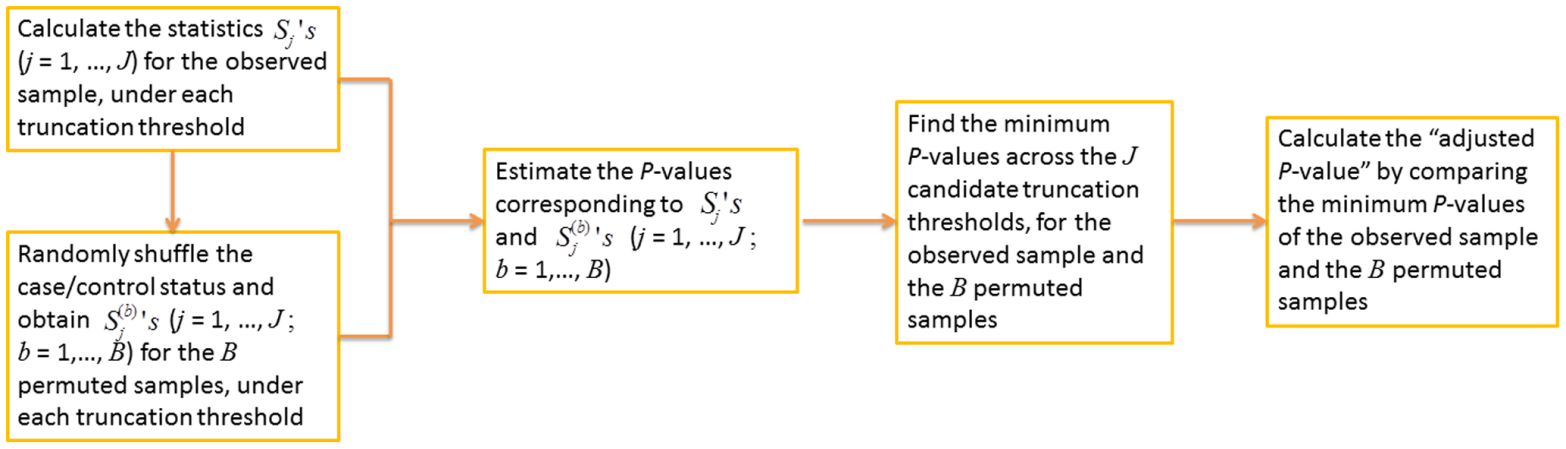
The workflow diagram of the *ADA* method.

With a total of *B* permutations, we can estimate the *P*-value of 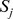 for the observed sample as 
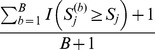
, for each truncation threshold (*j* = 1, …, *J*). The *P*-value of 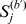 for the 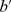
th permutation is estimated by 
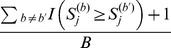
, for *j* = 1, …, *J* and 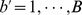. We can then find the minimum *P*-value 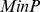 across the *J* candidate truncation thresholds for the observed sample, and the minimum *P*-value 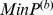 for the *b*th permuted samples (*b* = 1,…, *B*). For the observed and permuted samples, 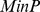 and 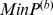 (*b* = 1,…, *B*) are *P*-values obtained from the “optimal” truncation thresholds that yield the most significant results (or, the minimum *P*-values) across candidate truncation thresholds. These “optimal” thresholds may vary across permuted samples, in order to preserve the validity of the proposed method. We then compare 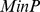 with 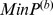 (*b* = 1,…, *B*) to assess the significance of the observed sample. The “adjusted *P*-value” is calculated by 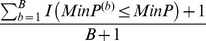. This method is referred to as “*ADA*”, because the per-site *P*-values of variant sites are combined adaptively. Figure 1 is a workflow diagram of the *ADA* method.

### Simulation Study

With the Cosi program [27], we first generated 200 data sets, each containing 10,000 chromosomes of 1 Mb regions. The Cosi program is based on the coalescent population genetic model [28] and is widely used to simulate human genome sequences. The chromosomes were generated according to the linkage disequilibrium patterns of the HapMap CEU (Utah residents with ancestry from northern and western Europe) samples. We randomly specified 25% of the variants with population MAF<1% to be causal variants. A region containing *d* causal variants was randomly selected as the causal region, where *d* = 3, 5, 10, 15, or 20. On average, a causal region spanned ∼3.6, ∼6.4, ∼12.8, ∼19.2, and ∼25.6 kb, for *d* = 3, 5, 10, 15, and 20, respectively. The numbers of neutral variants were ∼60, ∼100, ∼200, ∼300, ∼400, for the regions spanning ∼3.6, ∼6.4, ∼12.8, ∼19.2, and ∼25.6 kb, respectively. Across the 200 simulated data sets, the proportions of causal variants among all non-synonymous variants ranged from ∼4% to ∼8%. We randomly assigned *r_isk_* % of the *d* causal variants as deleterious variants, and let the remaining 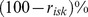 causal variants be protective variants. The value of *r_isk_* was set at 5, 20, 50, 80, and 100, respectively. In this way, we considered the simulation settings with mixtures of deleterious and protective variants. The population attributable risk (PAR) of each causal variant was specified at 0%, 0.1%, …, 0.5%, respectively.

Following the simulation setting of previous studies [3], [29]–[31], the genotype relative risk (GRR) of the *j*th causal variant is:
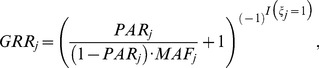
(3)where 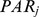 and 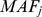 are the PAR and the population MAF of that variant, respectively. The indicator function 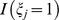 is 1 if the *j*th causal variant is protective, and is 0 if deleterious. Figure S1 shows the distributions of population MAFs and GRRs of the causal variants in our 200 simulated data sets. Because we focused on the detection of rare causal variants, the population MAFs of the causal variants were all smaller than 1% in our simulation. To generate the genotypes of a subject, we randomly selected two chromosomes from the pool of 10,000 chromosomes. The disease status of a subject with chromosomes 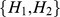 was determined by
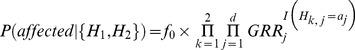
(4)
[29]–[31], where 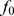 was the baseline penetrance, and 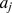 was the minor allele at the *j*th causal variant site. Following Cheung et al. [14], 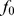 was specified at 1%, and the sample size was set at 1000. Pairs of chromosomes were drawn from the chromosome pool with replacement until 500 cases and 500 controls were sampled.

### Tests under Comparison

We compared *ADA* with 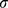 *-MidP* [14], burden tests, and non-burden tests. Cheung et al.'s [14] R script was used to implement their 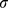 *-MidP* method (http://www.columbia.edu/~sw2206/softwares.htm). We followed the default of the 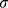 *-MidP* R script, single-nucleotide polymorphisms with MAF>5% in the combined sample of cases and controls were excluded from the analyses of 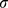 *-MidP* and *ADA*. To have a fair comparison between these two methods, the *P*-values used in Equations (1) and (2) (i.e., 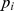
's) are obtained by the mid *P*-values according to the Fisher's exact test [14], [26].

Four burden tests including the fixed-threshold approach with MAF thresholds of 1% and 5% (i.e., “*T1*” and “*T5*”, respectively) [4], the weighted-sum approach (i.e., “*WS*”) [3], and the variable-threshold approach (i.e., “*VT*”) were implemented with the R script by Price et al. [5] (http://genetics.bwh.harvard.edu/rare_variants/). Because *VT* needs permutations to get *P*-values, Price et al. [5] performed permutations for all the four tests (*VT*, *WS*, *T1*, and *T5*) in their R script, at almost no extra computational cost. Note that the original *VT* script performs right-tailed tests for all the four methods, and therefore they are underpowered when 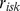 is low. We modified the original *VT* script to perform two-tailed tests and used the revised R script to implement the four burden tests.

Two non-burden tests including the sequence kernel association test (i.e., “*SKAT*”) [7] and the optimal test (i.e., “*SKAT-O*”) [8] that optimally combines the burden tests and *SKAT* were implemented with the R package “SKAT” [32]. We used the default weight function in the package “SKAT”, 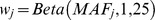, as the weight given to the *j*th variant site with MAF of 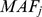.

The *P*-values of *ADA*, 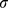 *-MidP*, *VT*, *WS*, *T1*, and *T5* were obtained with 10,000 permutations when evaluating the type-I error rates and 1,000 permutations when evaluating power, respectively. For *SKAT* and *SKAT-O*, we used the default method in the package “SKAT” to compute *P*-values, which was an exact method that computed *P*-values by inverting the characteristic function of the mixture chi-square distribution [33].

## Results

### Type-I Error Rates

By setting the PAR at exactly 0% and using ∼25.6 kb regions, we evaluated type-I error rates by performing 1,000 replications for each of the 200 simulated data sets. Based on the 200,000 ( = 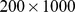) replications across the 200 simulated data sets, Table 1 shows that all of the eight tests are valid in the sense that their type-I error rates match the nominal significance levels.

**Table 1:** Type-I error rates.

nominal significance level	0.0001	0.005	0.010	0.015	0.020	0.025	0.030	0.035	0.040	0.045	0.050
*SKAT-O*	0.0001	0.0054	0.0102	0.0151	0.0196	0.0246	0.0295	0.0347	0.0396	0.0444	0.0492
*SKAT*	0.0001	0.0048	0.0096	0.0142	0.0191	0.0237	0.0288	0.0337	0.0384	0.0434	0.0482
*σ-MidP*	0.0001	0.0050	0.0101	0.0149	0.0199	0.0248	0.0298	0.0348	0.0398	0.0448	0.0498
*ADA*	0.0001	0.0050	0.0100	0.0148	0.0199	0.0247	0.0297	0.0351	0.0400	0.0451	0.0500
*T1*	0.0001	0.0046	0.0096	0.0146	0.0196	0.0245	0.0294	0.0346	0.0399	0.0449	0.0501
*T5*	0.0001	0.0046	0.0098	0.0149	0.0198	0.0247	0.0296	0.0346	0.0398	0.0449	0.0498
*WS*	0.0001	0.0052	0.0103	0.0153	0.0204	0.0254	0.0304	0.0356	0.0402	0.0452	0.0502
*VT*	0.0001	0.0050	0.0100	0.0150	0.0201	0.0250	0.0302	0.0352	0.0404	0.0453	0.0503

### Power Comparisons

**Figure 2:**
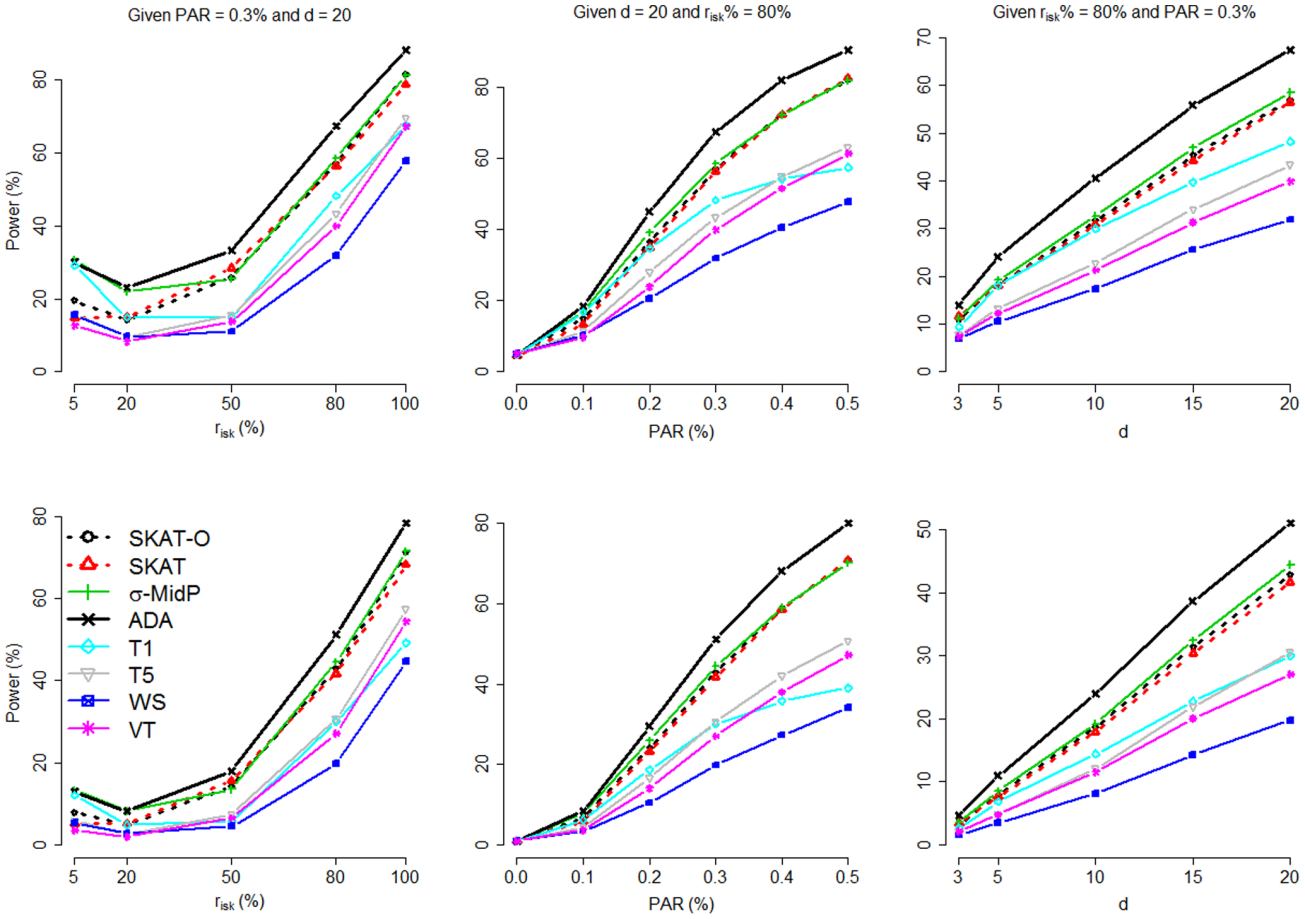
Comparison of power by *r_isk_* (the percentage of deleterious variants among the *d* causal variants), PAR, and *d* (the number of causal variants). The figure shows the power comparison by *r_isk_* (left column, given PAR = 0.3% and *d* = 20), PAR (middle column, given *d* = 20 and *r_isk_* = 80%), and *d* (right column, given *r_isk_* = 80% and PAR = 0.3%). The nominal significance level was set at 0.05 (top row) and 0.01 (bottom row), respectively.

When we evaluated power, a total of 100 replications were performed under each scenario (each combination of 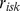, PAR, and *d*) for each of the 200 simulated data sets. Figure 2 presents the power averaged over the 200 data sets, where 100 replications were performed for each data set. Each point represents the result averaged from 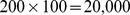 replications performed for some combination of 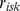, PAR, and *d*. The nominal significance level was set at 0.05 (top row) and 0.01 (bottom row), respectively. In the first column of Figure 2, power was assessed with a varying 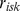, a fixed PAR (0.3%), and a fixed *d* (20).

Note that the lowest power occurs around 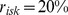 (among the five values of 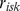), rather than 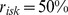 (the first column of Figure 2). This is because, in our simulation setting (following [29]), a deleterious variant has a larger effect size than a protective variant, given that they have the same MAF. For simplicity of illustration, we consider only one causal variant site. The probability that a subject has two rare variants at this site is extremely small and thus can be ignored. Equation (4) can be simplified as
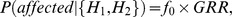
where 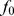 is the baseline penetrance and *GRR* is the genotype relative risk of the causal variant. Based on Equation (3),
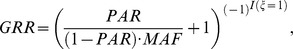
where the subscripts have been removed for simplification. Let 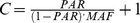. For case-control studies, the odds ratio (OR) of being affected among subjects who have a causal variant versus those who do not is an appropriate measure for effect size. Let 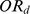 be the OR of being affected among subjects who have a deleterious variant versus those who do not. We have
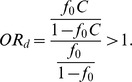
Let 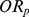 be the OR of being affected among subjects who have a protective variant versus those who do not. We have
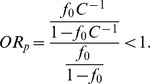
Because 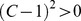,
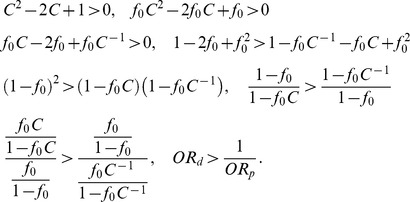
Thus, in our simulation setting (following [29]), a deleterious variant has a larger effect size than a protective variant, given that they have the same MAF. This is why the lowest power occurs at 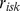 smaller than 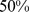.

In the second column, PAR varied, while *d* was fixed at 20 and *r_isk_* % was fixed at 80%. The setting of *r_isk_* % (80%) was chosen because regulatory sequences are likely to contain many more deleterious variants than protective variants [34], [35]. As for the third column, power was compared while *d* was varying, but *r_isk_* % was fixed at 80%, and PAR was fixed at 0.3%. *ADA* test showed the best performance under the majority of simulation scenarios.

### Application to Data from Dallas Heart Study

We applied the eight tests to a population-based resequencing study for the *ANGIOPOIETIN–LIKE 4* (*ANGPTL4*) gene [24], [25]. To learn the role of *ANGPTL4* in plasma triglyceride levels, Romeo et al. [24], [25] sequenced seven exons and the intron-exon boundaries of *ANGPTL4*. The important confounders when investigating plasma triglyceride levels include ethnicity, age, sex, and body-mass index (BMI) [24]. To remove the potential influence of ethnicity on triglyceride, we only analyzed the 1,045 European Americans from the total 3,551 subjects sampled from Dallas County residents [36]. The log-transformed triglyceride levels were adjusted for age, sex, and BMI, with a linear regression. The regression residuals were treated as new phenotypes that have been adjusted for important confounders. Subjects with residuals larger than the 70^th^ percentile and smaller than the 30^th^ percentile were treated as cases and controls, respectively. Then the subjects with missing genotypes were removed from our analysis. Finally, we had 179 cases and 213 controls (the numbers of cases and controls were not necessarily equal, because we removed the subjects with missing genotypes after marking the 30^th^ and 70^th^ percentiles of the phenotype).

We then applied the eight tests to this data set. The variants with MAF<5% in the *ANGPTL4* gene were analyzed to test for their associations with triglyceride. The significant association of *ANGPTL4* with triglyceride was previously reported by other investigators [14], [37]. With a significance level of 0.05, the four burden tests (*VT*, *WS*, *T1*, and *T5*) did not show significant association of *ANGPTL4* with triglyceride, whereas the other four tests including *ADA*, *SKAT*, *SKAT-O*, and 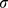 *-MidP* confirmed this association (see Table 2).

**Table 2:** Analysis of the Dallas Heart Study data.

	*SKAT-O*	*SKAT*	*σ-MidP* a	*ADA* a	*T1* a	*T5* a	*WS* a	*VT* a
*P*-value	0.024	0.012	0.028	0.011	0.584	0.070	0.184	0.486

^a^ *P*-values were estimated based on 10^4^ permutations.

## Discussion

In this work, we have proposed a powerful *ADA* method for rare causal variants detection. Instead of fixing a threshold to truncate *P*-values, we recommend searching for the “optimal” threshold from among multiple candidate truncation thresholds. The validity of *ADA* is preserved because we allow the permuted and observed data to have different “optimal” truncation thresholds. Here, we use 11 candidate *P*-value truncation thresholds, 0.10, 0.11, 0.12, …, 0.20. We do not consider a more stringent threshold (<0.10), because testing for a single rare variant is usually underpowered [2], [20]–[22] and a stringent threshold may exclude the information of causal variants. We neither consider a more liberal threshold (>0.20), because that may include more noise from neutral variants. To show this, we also evaluated the *ADA* method with 21 candidate *P*-value truncation thresholds (0.05, 0.06, 0.07, …, 0.25). Table 3 lists the power of the *ADA* method with two sets of candidate *P*-value truncation thresholds. Using 21 candidate *P*-value truncation thresholds (0.05, 0.06, 0.07, …, 0.25) does not contribute a noticeable power gain to *ADA*.

**Table 3:** Power (%) of the *ADA* method with two sets of candidate *P*-value truncation thresholds.

candidate *P*-value truncation thresholds	Given PAR = 0.3% and *d* = 20					Given *d* = 20 and *r_isk_* = 80%						Given *r_isk_* = 80% and PAR = 0.3%				
	*r_isk_* (%)					PAR (%)						*d*				
	5	20	50	80	100	0.0	0.1	0.2	0.3	0.4	0.5	3	5	10	15	20
Nominal significance level = 5%																
0.10, 0.11,… 0.20	29.97	23.17	33.28	67.41	88.24	4.84	18.45	45.06	67.41	82.03	90.47	14.00	24.16	40.58	55.80	67.41
0.05, 0.06,…, 0.25	29.38	23.50	35.04	68.73	89.31	5.04	18.56	46.09	68.73	83.60	91.91	14.64	25.50	42.24	57.30	68.73
Nominal significance level = 1%																
0.10, 0.11,…, 0.20	13.00	8.17	17.99	51.10	78.32	1.00	8.39	29.50	51.10	68.09	80.03	4.68	10.99	24.01	38.65	51.10
0.05, 0.06,…, 0.25	12.25	8.22	18.74	51.98	79.17	0.93	8.46	30.03	51.98	69.45	81.22	4.88	11.50	24.93	39.59	51.98

Note that the statistic, 
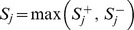
, is the maximization of the score accumulated by deleterious-inclined variants and that accumulated by protective-inclined variants. Another justifiable statistic is 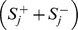, which is more powerful than *ADA* when the numbers of deleterious and protective variants are comparable, but it is less powerful when the region contains more deleterious variants than protective variants (or, more protective variants than deleterious variants). Because both evolutionary mechanisms and empirical studies support the hypothesis that regulatory sequences contain substantial amounts of weakly deleterious variation [34], [35], [38], [39], the number of deleterious variants may surpass that of protective variants in most situations. Therefore, we still advocate using 
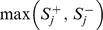
, rather than 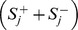.

The computation time of *ADA* is slightly longer than that of 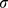 *-MidP*. For simulated data sets each containing 500 cases and 500 controls in ∼3.6 kb regions (include ∼60 nonsynonymous variant sites), 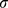 *-MidP* (http://www.columbia.edu/~sw2206/softwares.htm) with 1000 permutations on average needs ∼27.8 sec, *ADA* with 1000 permutations needs ∼28.6 sec, *SKAT-O* needs ∼6.7 sec, while *VT* with 1000 permutations takes only ∼0.9 sec. When the region was enlarged to ∼6.4 kb (include ∼110 nonsynonymous variant sites), 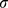 *-MidP* with 1000 permutations on average needs ∼45.3 sec, *ADA* with 1000 permutations needs ∼45.9 sec, *SKAT-O* needs ∼9.2 sec, while *VT* with 1000 permutations takes 1.2 sec. These were measured on a Linux platform with an Intel Xeon E5-2690 2.9 GHz processor and 2 GB memory. Although the computation time of *VT* or *SKAT-O* is much shorter than that of *ADA* (or 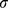 *-MidP*), the power of *VT* or *SKAT-O* is not comparable to *ADA*.

Rare causal variants are likely to play an important role in the etiology of some complex diseases [40]–[45], but they are difficult to detect by single-locus tests [2], [20]–[22]. Grouping variant sites in a functional region and testing for association with an omnibus statistic is a promising strategy. Compared with the burden tests (*VT*, *WS*, *T1*, and *T5*) and the non-burden tests (*SKAT* and *SKAT-O*) evaluated here, *ADA* is more robust to the inclusion of neutral variants. With the advancement in next-generation sequencing technology, all single-nucleotide variants (causal or neutral) can be sequenced. *ADA* is recommended for its ability to guard against the noise of neutral variants.

## Supporting Information

Figure S1 **The distributions of the population minor allele frequencies (MAFs) and genotype relative risks (GRRs) of the causal variants in our 200 simulated data sets.**
(TIFF)
