# Supplementary figures and images for: Rare Variant Association Testing by Adaptive Combination of P-values
Source: PLoS One. 2014 Jan 15;9(1):e85728. doi: 10.1371/journal.pone.0085728 (PMC3893264; doi:10.1371/journal.pone.0085728)

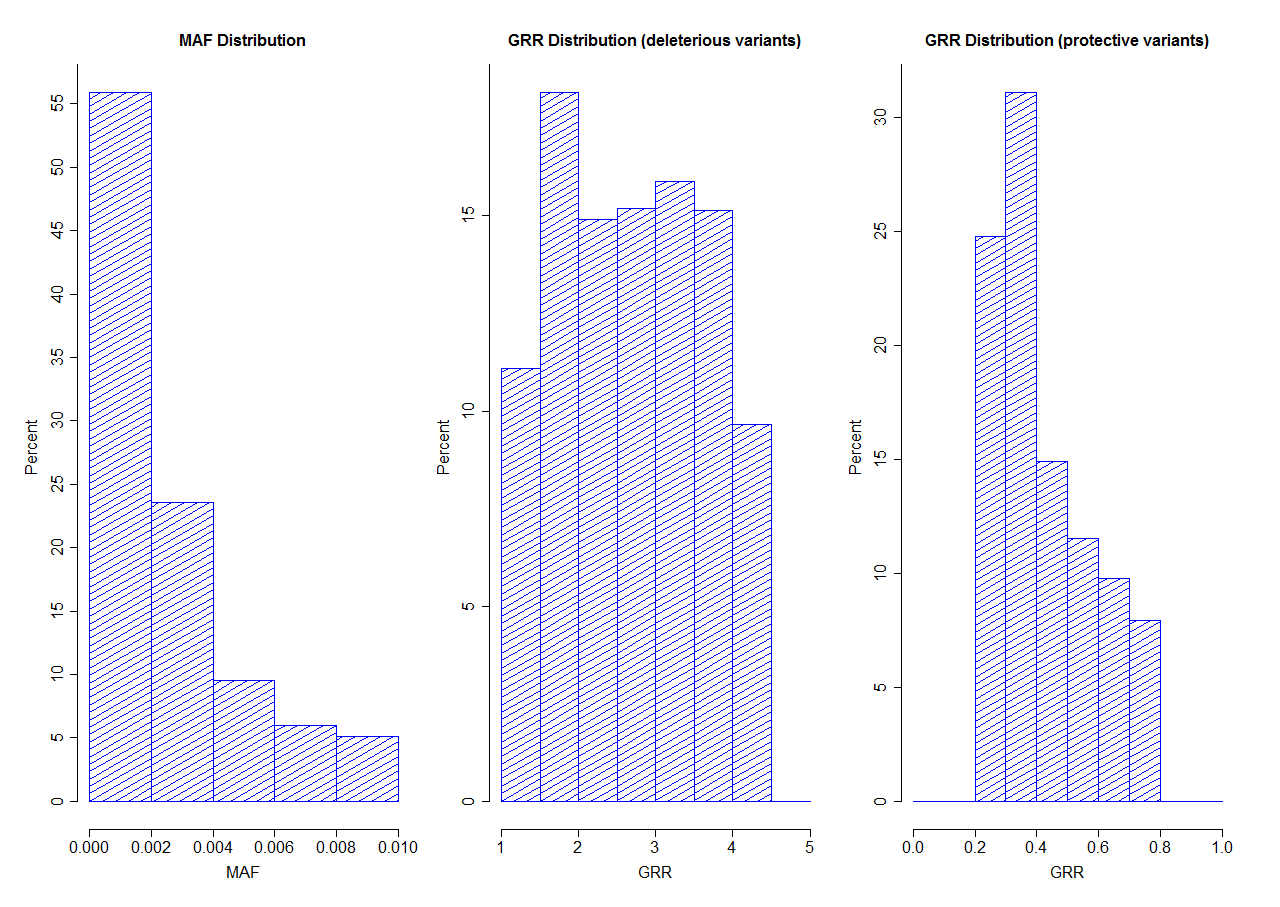

Supplement: Figure S1 — The distributions of the population minor allele frequencies (MAFs) and genotype relative risks (GRRs) of the causal variants in our 200 simulated data sets. (TIFF) [file pone.0085728.s001.tiff]
